# CircPHGDH downregulation decreases papillary thyroid cancer progression through miR-122-5p/PKM2 axis

**DOI:** 10.1186/s12885-024-12199-5

**Published:** 2024-04-23

**Authors:** Jiying Shen, Zhirong Ma, Jin Yang, Tianzhen Qu, Yu Xia, Yingjie Xu, Ming Zhou, Weiwei Liu

**Affiliations:** Department of General Surgery, Shanghai Tongren Hospital, Shanghai Jiao Tong University School of Medicine, 1111 Xianxia Road, 200336 Shanghai, China

**Keywords:** circPHGDH, miR-122-5p, PKM2, Papillary thyroid cancer, Aerobic glycolysis

## Abstract

**Background:**

Although papillary thyroid carcinoma (PTC) has a favorable prognosis, it could affect patient life quality and become a serious threat because of invasion and metastasis. Many investigations have suggested that circular RNAs (circRNAs) are involved in different cancer regulations. Nevertheless, circRNAs role in invasive PTC remains unclear.

**Methods:**

In the present investigation, next-generation sequencing was applied to explore abnormal circRNA expression. The expression of circRNA phosphoglycerate dehydrogenase (circPHGDH) in PTC cell lines and tissues were examined. Then, we investigated regulatory mechanism and circPHGDH downstream targets using bioinformatics analysis and luciferase reporting analysis. Then transwell migration, Cell Counting Kit-8 (CCK8) and 5-ethynyl-2’-deoxyuridine (EdU) assays were used for cells migration and proliferation analysis. In vivo metastasis and tumorigenesis assays were also employed to evaluate the circPHGDH role in PTC.

**Results:**

The data showcased that circPHGDH expression increased in both PTC cell lines and tissues, which suggested that circPHGDH functions in PTC progression. circPHGDH downregulation suppressed PTC invasion and proliferation in both in vivo and in vitro experiments. Bioinformatics and luciferase reporter results confirmed that both microRNA (miR)-122-5p and pyruvate kinase M2 subtype (PKM2) were downstream targets of circPHGDH. PKM2 overexpression or miR-122-5p suppression reversed PTC cell invasion and proliferation post silencing circPHGDH by restoring aerobic glycolysis.

**Conclusion:**

Taken together, our research found that circPHGDH downregulation reduced PTC progression via miR-122-5p/PKM2 axis regulation mediated by aerobic glycolysis.

## Introduction

Papillary thyroid carcinoma (PTC) is among the most popular malignant tumors, which results in ∼ 85% all thyroid cancers [1]. Although 10-year survival rates for PTC patients may reach over 90% due to good postoperative prognosis and slow tumor growth [2–3], many PTC patients have poor prognosis because of lymph node as well as distant metastasis [4]. Therefore, it is very important to improve our knowledge of PTC pathogenesis in order to find novel therapeutic PTC targets.

CircRNA is non-coding RNA that newly discovered. Studies have revealed that circRNAs play important roles as regulators of tumorigenesis because of their covalently closed circular structure, without 5ʹ cap or 3ʹ poly A tail [5]. It has also been discovered that many circRNAs take part in PTC progression, such as circ-0062389, hsa_circ_0039411, hsa_circ_0001666, circ-0058124 and circ-ITCH [6–10]. In addition, circRNAs can regulate the downstream target mRNA translations by sponging miRNAs [11–13].

Investigations also showcased that tumor cells have higher glucose consumption and increased lactate production rates in a low oxygen microenvironment compared with normal conditions [14]. More research have discovered that aerobic glycolysis in solid tumors promotes malignant progression, including that of PTC [15, 16]. Aerobic glycolysis is a promising cancer therapeutic intervention because it provides cancer cells with sufficient biomass energy, the building material for cell growth and proliferation, as well as the advantage of adapting to a high-oxidation microenvironment for better survival [17]. Whether circRNAs can regulate PTC progression by influencing aerobic glycolysis is still unclear.

This study found that the circRNA regulating phosphoglycerate dehydrogenase (circPHGDH) in PTC was highly expressed. Downregulation of circPHGDH decreased PTC cell proliferation and migration. Our investigations also confirmed that circPHGDH expression enhanced aggressive PTC cell progression via miR-122-5p/pyruvate kinase M2 subtype (PKM2)-mediated glycolysis regulation. Our data reveals a new insight into the involvement and molecular mechanisms underlying the circPHGDH actions in PTC.

## Materials & methods

### Patients and specimens

Our lab harvested PTC samples and matched noncancerous samples (*n* = 10) from PTC patients diagnosed at Tongren Hospital, Shanghai Jiao Tong University School of Medicine. No patients had undergone chemotherapy or radiotherapy in prior surgeries. The study was approved by the Ethics Committee Board of Tongren Hospital, Shanghai Jiao Tong University School of Medicine. All study subjects gave written informed consent for this study.

### Fluorescence in situ hybridization

We designed a specific probe to circPHGDH (Dig-5′-GTAAGCTGGGCACAGACTGTGAAGGCC-3′-Dig) which was manufactured by Geneseed Biotech, Guangzhou, China. The signals were obtained by Cy3-conjugated anti-biotin antibodies (Jackson ImmunoResearch Inc., West Grove, PA, USA). Cell nuclei were counterstained using 4,6-diamidino-2-phenylindole (DAPI, #28718-90-3, Sigma-Aldrich, St. Louis, MO, USA).

### Cell culture

Human thyroid epithelial cell line Nthy-ori 3 − 1 and PTC cell lines including SW579, TPC-1, K1 and FTC-133 were purchased from Chinese Academy of Sciences (Shanghai, China). We cultured cells at 37 °C in 5% CO_2_ atmosphere using DMEM containing 10% fetal bovine serum (#Sv30087.01, HyClone Laboratories Inc., USA).

### siRNA and miRNA mimic

Our team designed miR-122-5p mimics and circPHGDH silencing (si)RNA that was then manufactured by Genepharma (Suzhou, China). The PKM2 overexpression vector was constructed by inserting cDNA from PKM2 into the pcDNA3.1 vector. Lipofectamine 2000 was used for transfection following instructions of the manufacturer (#11,668, Invitrogen, Carlsbad, CA, USA).

### Cell proliferation assay

We seeded a total of 2 × 10^3^ TPC-1 or SW579 cells per well. At established times, every sample absorbance at 450 nm was captured utilizing Cell Counting Kit-8 (CCK8) assays (#40203ES60, Yeasen Biotech Co., Ltd, Shanghai, China). The viability curves of the cells were then plotted and analyzed.

### 5-ethynyl-2′-deoxyuridine (EdU) assay

EdU assay kits (#C00003, RiboBio, Guangzhou, China) were employed to investigate cell DNA synthesis and proliferation. A total of 10,000 SW579 or TPC-1 cells were seeded in 96-well plates and incubated over the night. EdU solution (25 µM) was added to plates, which were incubated for another 1 d. Cells were fixed in 4% formalin for 2 h at room temperature. We then permeabilized cells in 0.5% Triton X-100 for 10 min, followed by addition of 200 µL Apollo reaction solution to stain the EdU-labeled DNA and 200 µL DAPI to stain cell nuclei. Cell proliferation and DNA synthesis were quantified using Nikon microscope (Nikon, Tokyo, Japan).

### Transwell migration assay

Our lab adjusted transfected cells for 48 h to with 2.0 × 10^5^/mL dose. Briefly, we applied 200 µL/well cell suspension to upper wells of Transwell chambers (#PI8P01250, Millipore, Billerica, MA, USA). Meanwhile, 500 µL of medium including 10% FBS was put to lower wells. After a 1-day incubation, we fixed invasive cells on the bottom side of the upper wells with methanol for a quarter to stain them with crystal violet for 20 min. We observed penetrating cells using a microscope and counted the invading cell numbers in five randomly chosen fields of view for every sample.

### Quantitative reverse transcription-PCR (qRT-PCR) analysis

Our lab extracted total RNA from tumor tissues or cells utilizing TRIzol Reagent (#15,596,026, Invitrogen, Carlsbad, CA, USA) before using PrimeScript™ RT Reagent kit (#RR037Q, Takara Bio, Inc. Japan) for cDNA synthesis. The cDNAs were then subjected to qRT-PCR detection with U6 and GAPDH primers applied as internal controls utilizing 2^−ΔΔCq^ method. Primers applied to assay circPHGDH expression contained forward, 5′-GCTTGGGGTCTAGGTAAG-3′ and reverse, 5′-GTGGCAGAGCGAACAATAAG-3′. miR-122-5p primers were forward, 5′-TATTCGCACTGGATACGACACAAAC-3′; reverse: 5′-GCCCGTGGAGTGTGACAATGGT-3′. *PKM2* primers were forward, 5′-ATTATTTGAGGAACTCCGCCGCCT-3′ and reverse, 5′-ATTCCGGGTCACAGCAATGATGG-3′; U6 primers were forward, 5′-CTCGCTTCGGCAGCACA-3′; reverse: 5′-AACGCTTCACGAATTTGCGT-3′; GAPDH primers were forward, 5′-AATGGGCAGCCGTTAGGAAA-3′; reverse: 5′-TGAAGGGGTCATTGATGGCA-3′.

circPHGDH, miR-122-5p and PKM2.

### Glucose consumption and lactate production assay

Our team employed lactate assay kits (#MAK065, Sigma, St. Louis, MO, USA) and glucose assay kits (#GAHK-20, Sigma, St. Louis, MO, USA) for lactate production and glucose consumption detection, as previously described [18].

### Target prediction and dual luciferase reporter test

Our team predicted interactions among circPHGDH, miR-122-5p and PKM2 utilizing Starbase database. Fragment sequences containing potential binding sites were inserted into pmirGLO vector (#E2261, Promega, Madison, WI, USA). Then, miR-122-5p mimics/negative control (NC) and pmirGLO vector were transfected into cells utilizing Lipofectomine 2000. We measured luciferase intensity employing a Dual Luciferase Assay Kit (#11402ES60, Promega, Madison, WI, USA).

### In vivo experiments

Four-week-old BALB/c nude mice weighing 15–20 g (SLARC, Shanghai, China) were used. All animal experiments were approved by the Ethics Committee of Tongren Hospital, Shanghai Jiao Tong University School of Medicine. To establish a nude mouse model, we injected short hairpin (sh)-NC or sh-circPHGDH TPC-1 cells (2 × 10^6^ in 100 µl PBS) expressing into nude mice flanks. Both tumor volumes and weights were calculated. All mice were sacrificed by neck dislocation.

For tumor metastasis analysis, we tail intravenous injected luminescence-labeled TPC-1 cells (luc-TPC-1) (2 × 10^5^ in 100 µl PBS) stably transfected with sh-circPHGDH or sh-NC into nude mice tail veins. After 4 weeks, lung metastasis was evaluated using a bioluminescence imaging system. We utilized Hematoxylin/eosin (H/E) staining to capture metastatic foci in lung tissues. All mice were sacrificed by neck dislocation.

### Immunohistochemistry

We fixed tumor tissues in 4% formaldehyde, which we embedded in paraffin to cut into 4-µm thick sections. Ki67 (1:1000) was utilized as primary antibody before application of secondary antibody (1:2000). Technician used DAPI to counterstain nuclei. We evaluated sections via optical microscopy. For H/E staining, we stained fixed lung tissue sections by H/E dye solution.

### Statistical analysis

A statistician performed analyses applying GraphPad Prism (La Jolla, CA, USA). Tukey’s multiple comparison and one-way variance analysis tests were applied for multiple group comparisons. P-value < 0.05 was considered as statistical significance.

## Results

### CircPHGDH expression level in PTC tissues and cell lines

**Fig. 1:**
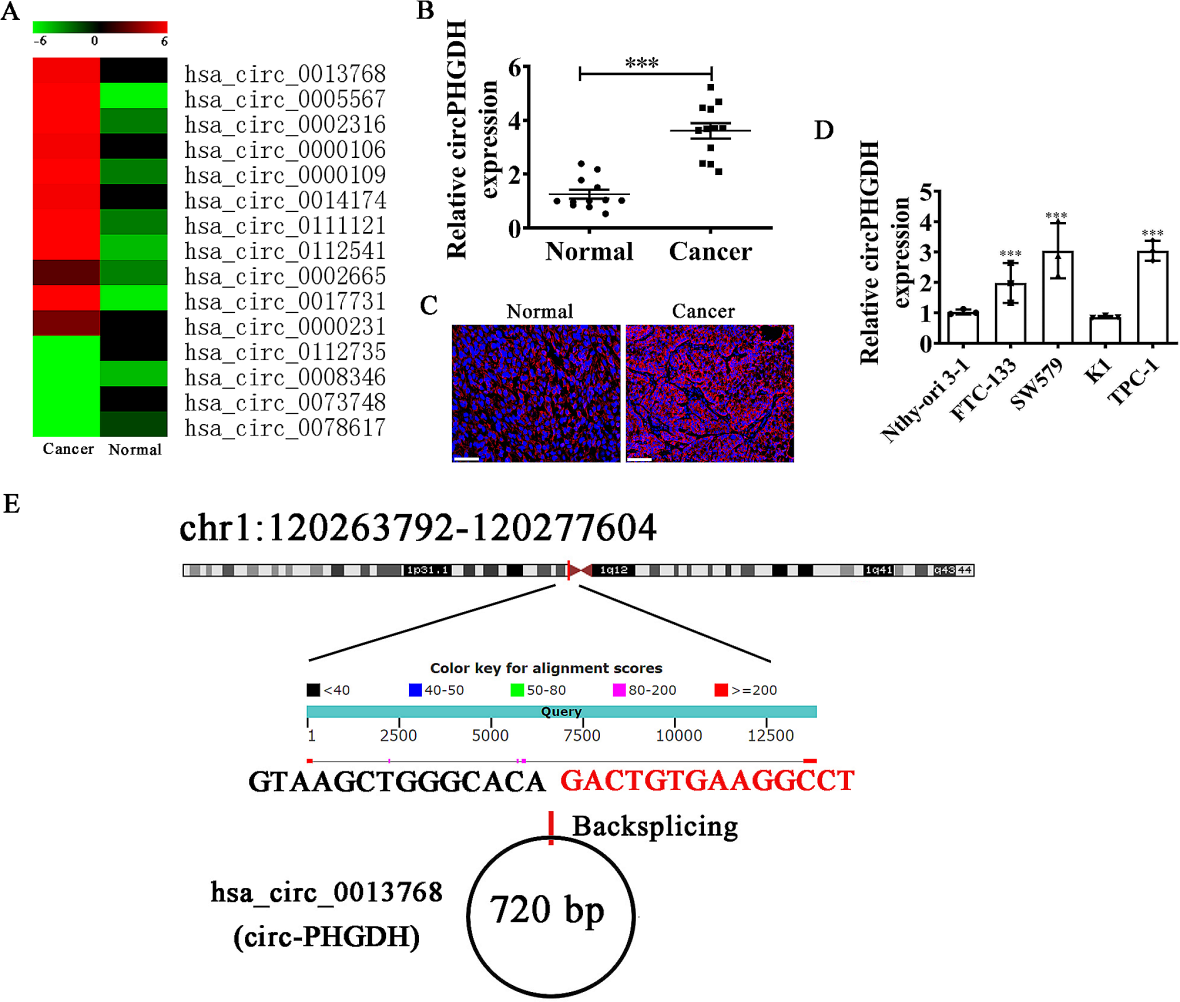
Relative expression of circPHGDH in PTC tissues and cell lines. (**A**) High-throughput sequencing detected show the abnormal expression of circRNA between PTC tissue and paired non-cancerous tissue. (**B**) RT-qPCR detection show the expression of circPHGDH (hsa_circ_0013768) in ten pair of PTC tissue and paired non-cancerous tissue. Data are presented as means ± SD. ^***^P < 0.001 vs. Normal. (**C**) FISH detection show the expression and subcellular distribution of circPHGDH in PTC tissue and paired non-cancerous tissue. Scale bar, 50 μm. (**D**) RT-qPCR detection show the expression of circPHGDH in PTC cell line FTC-133, SW579, K1, TPC-1 and a human thyroid epithelial cell line Nthy-ori 3 − 1. Data are presented as means ± SD. ^***^P < 0.001 vs. Nthy-ori 3 − 1. (**E**) The genomic loci of the *PHGDH* gene and circPHGDH.

Next-generation sequencing showcased abnormal circPHGDH expression comparing with PTC tissues and paired non-cancerous tissues. Differential expression of circPHGDH was observed in PTC tissues (Fig. 1A). qRT-PCR data indicated that circPHGDH expression significantly incremented in PTC tissues comparing with paired non-cancerous tissue (Fig. 1B). Fluorescence in situ hybridization results also demonstrated that circPHGDH expression increased in PTC tissues comparing with paired non-cancerous tissue. Meanwhile, the data showcased that circPHGDH was mainly distributed in cytoplasm (Fig. 1C). Furthermore, significantly higher circPHGDH expression was observed in PTC cells lines including K1, FTC-133, SW579 and TPC-1 when compared to normal human thyroid epithelial cell line Nthy-ori 3 − 1. In particular, both TPC-1 and SW579 cells showed the highest circPHGDH expression, which we selected for further study (Fig. 1D). Bioinformatics analysis revealed that circPHGDH was derived by cyclizing six exons from PHGDH gene located at chr1:120263792–120,277,604. PHGDH was 13,812 bp and spliced mature circRNA was 720 bp (Fig. 1E). The circRNA contained the same sequence as hsa_circ_0013768 and was consequently relabeled circPHGDH.

### CircPHGDH downregulation suppressed tumor growth and PTC proliferation in both in vitro and in vivo experiments

**Fig. 2:**
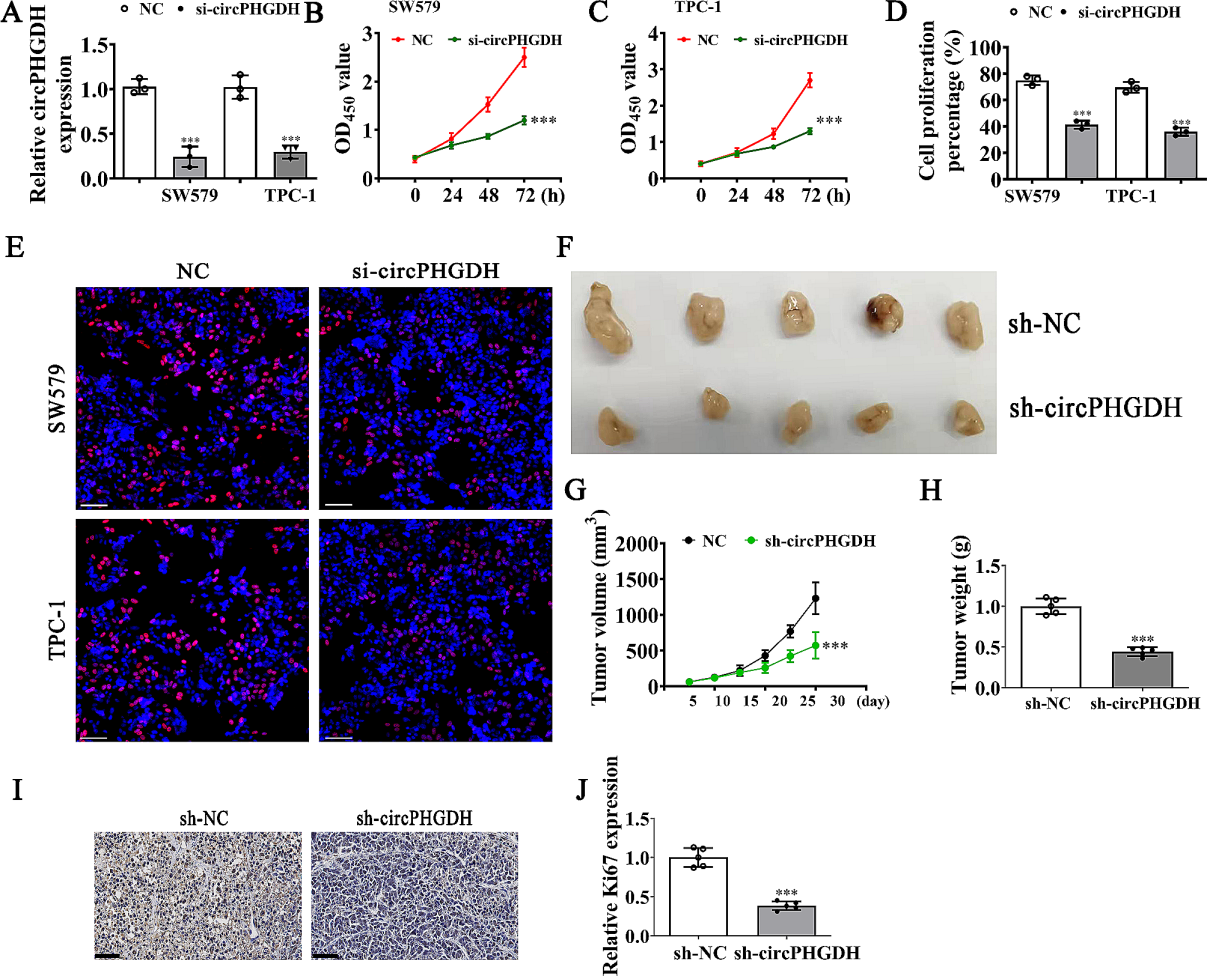
Downregulation circPHGDH suppressed PTC proliferation and tumor growth in both in vitro and in vivo experiment. (**A**) RT-qPCR detection show the expression of circPHGDH in both SW579 and TPC-1 cells after transfected with NC or si-circPHGDH. The data are expressed as the mean ± SD. ^***^p < 0.001 vs. NC. (**B** and **C**) CCK8 detection show the proliferation ability of SW579 and TPC-1 cells. The data are expressed as the mean ± SD. ^***^p < 0.001 vs. NC. (**D** and **E**) EdU assay show the proliferation of both SW579 and TPC-1 cells after transfected with NC or si-circPHGDH. The data are expressed as the mean ± SD. ^***^p < 0.001 vs. NC. Scale bar, 50 μm. (**F**) Representative photographs of TPC-1 tumor formation in the xenografts of nude mice. (**G**) Summary of the tumor volume in mice that were measured every five days. Data are presented as the mean ± SD. ^***^p < 0.001 vs. NC. (**H**) Tumor weight was measured at 25 days post-injection. Data are presented as the mean ± SD. ^***^p < 0.001 vs. NC. (**I** and **J**) Immunohistochemical analysis showing the percentage of Ki67-positive cells. Data are presented as the mean ± SD. ^***^p < 0.001 vs. NC. Scale bar, 50 μm

To investigate circPHGDH functions during PTC progression, we synthesized siRNAs targeting the circPHGDH back-spliced site to suppress circPHGDH expression. qRT-PCR detection showed that circPHGDH expression decreased significantly in TPC-1 and SW579 cells after transfection with siRNA against circPHGDH (Fig. 2A). CCK8 (Fig. 2B and C) and EdU (Fig. 2D and E) assays indicated that downregulation of circPHGDH suppressed PTC cell proliferation. In vivo tumorigenesis in xenografts of nude mice using TPC-1 cells showed that circPHGDH silencing significantly decreased tumor growth in both weight and volume (Fig. 2F–H). Immunohistochemical staining for Ki67 also confirmed that circPHGDH silencing inhibited Ki67 expression in tumor tissues (Fig. 2I and J), demonstrating that downregulation of circPHGDH inhibited PTC proliferation and tumor growth in in vitro and in vivo experiments.

### Downregulation of circPHGDH suppressed PTC cell migration and pulmonary metastasis

**Fig. 3:**
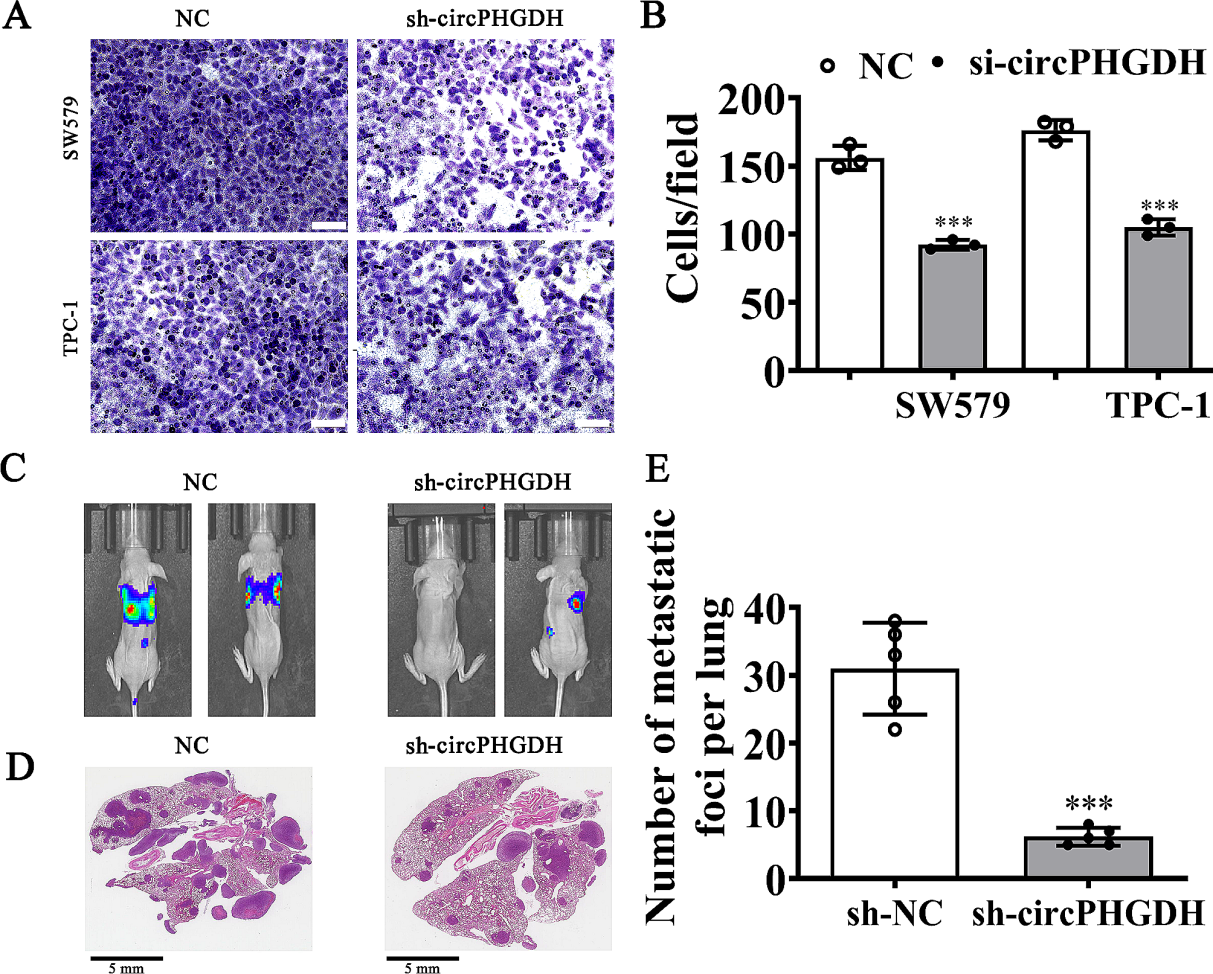
Downregulation circPHGDH suppressed PTC cells migration and pulmonary metastasis. (**A** and **B**) Transwell detection show the migration ability of both SW579 and TPC-1 cells after silence circPHGDH. The data are presented as the mean ± SD. ^***^p < 0.001 vs. NC. Scale bar, 50 μm. (**C**) Living imaging detection show the pulmonary metastasis ability of TPC-1 cells. (**D** and **E**) The numbers of metastatic foci in lung tissues were caculation according to the HE staining. The data are expressed as the mean ± SD. ^***^p < 0.001 vs. sh-NC

Transwell assays for migration showed that circPHGDH silencing inhibited migration of both TPC-1 and SW579 cells (Fig. 3A–B). Live imaging detection showed the pulmonary metastasis of luc-TPC-1 cells and revealed that circPHGDH silencing reduced pulmonary metastasis by decreasing the metastatic foci numbers in lung tissues, as was also shown by H/E staining analyses (Fig. 3C-E). These results suggested that downregulating circPHGDH suppressed PTC cell migration and pulmonary metastasis.

### Mir-122-5p and PKM2 were circPHGDH downstream targets

**Fig. 4:**
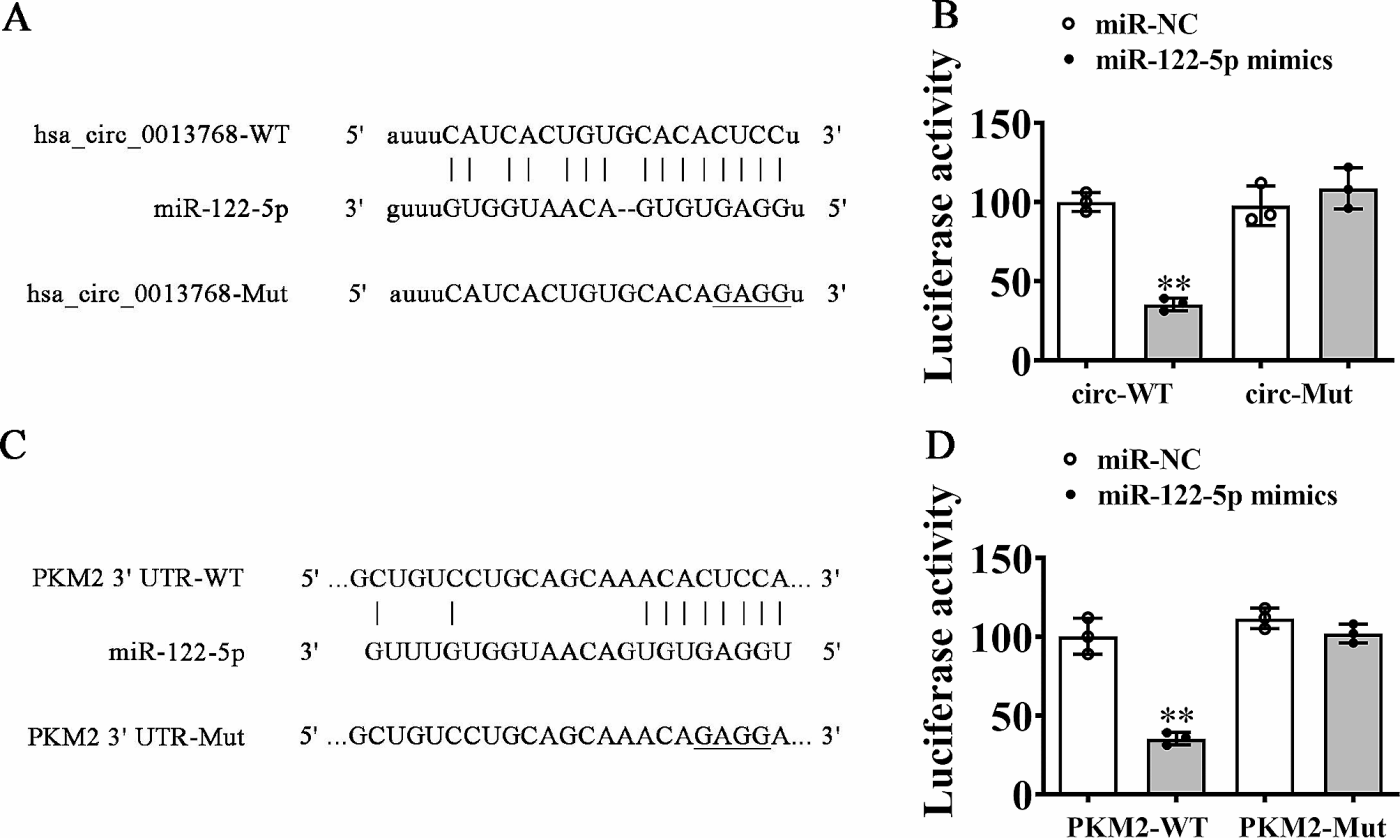
MiR-122-5p and PKM2 were the downstream target of circPHGDH. (**A**) Prediction of binding sites of miR-122-5p in circPHGDH. The MUT version of circPHGDH is presented. (**B**) Relative luciferase activity determined 48 h after transfection of HEK293T cells with miR-122-5p mimic/NC or circPHGDH WT/Mut. Data are presented as means ± SD. ^**^P < 0.01. (**C**) Prediction of binding sites of miR-122-5p within the 3’UTR of PKM2. The MUT version of 3’-UTR-PKM2 is shown. (**D**) Relative luciferase activity determined 48 h after transfection of HEK293T cells with miR-122-5p mimic/NC or 3’UTR-PKM2 WT/Mut. Data are presented as means ± SD. ^**^P < 0.01

Bioinformatics analyses found that circPHGDH can interact with a bunch of miRNAs. We then constructed luciferase reporter vector containing circPHGDH sequence and used it to confirm that miR-122-5p suppressed luciferase activity in wild-type (WT) yet not mutated (MUT) cell lines (Fig. 4A and B) saying that miR-122-5p was circPHGDH target. Bioinformatics data also indicated that PKM2 was miR-122-5p downstream target. To better validate the correlation between miR-122-5p and PKM2, we constructed MUT or WT 3’UTR-PKM2 sequences such like miR-122-5p binding sequence into luciferase reporter vectors (Fig. 4C). We transfected these luciferase reporter vectors into HEK293 cells with or not miR-122-5p mimic. Luciferase reporter analyses demonstrated that miR-122-5p suppressed luciferase activity in WT while not MUT cell lines (Fig. 4D) telling that PKM2 was miR-122-5p target.

### PKM2 overexpression or mir-122-5p suppression reversed PTC cell migration and proliferation after silencing circPHGDH

**Fig. 5:**
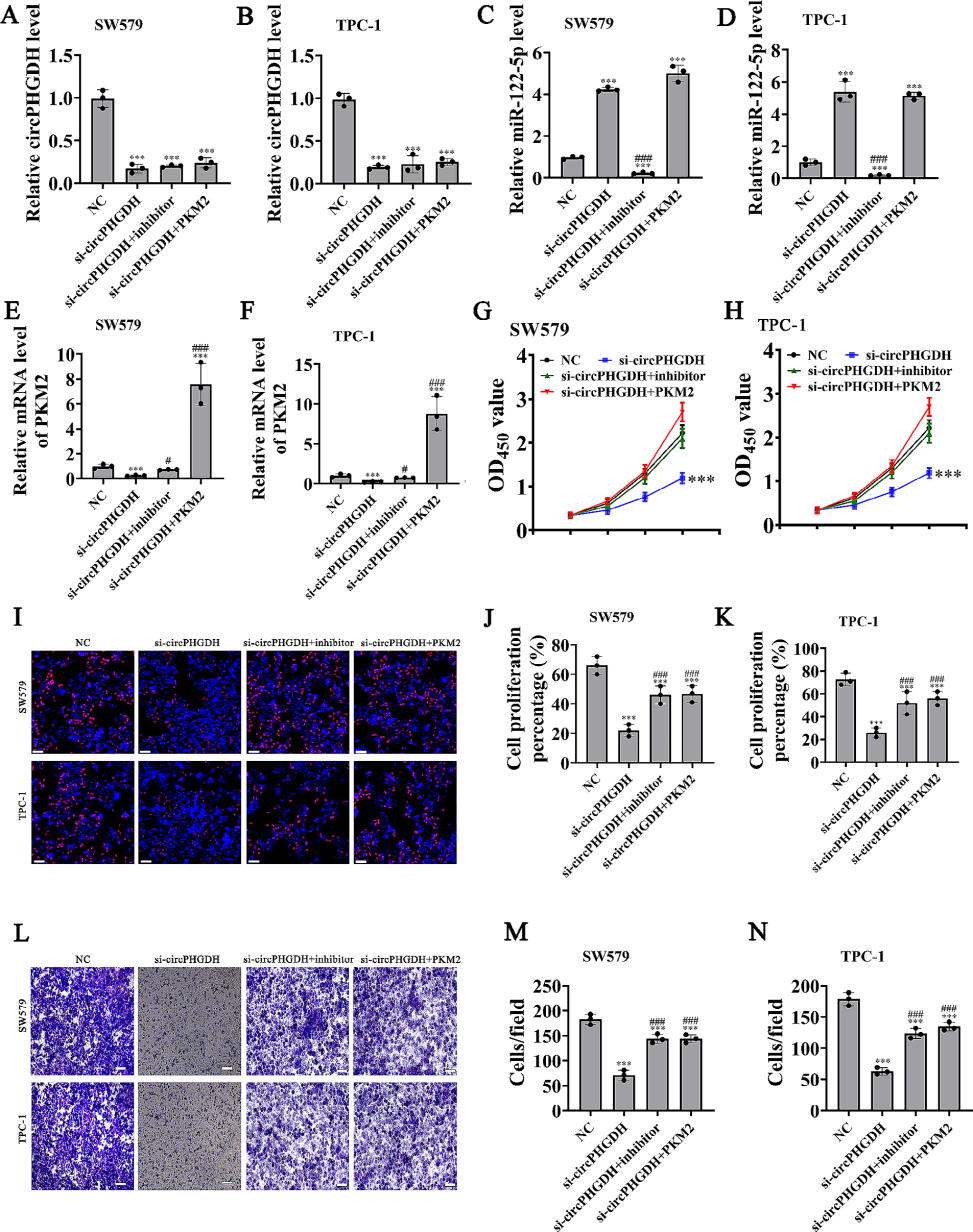
Overexpression of PKM2 or inhibit miR-122-5p reversed PTC cells proliferaion and migration after silence circPHGDH. (**A**-**F**) RT-qPCR detection showing the expression of circPHGDH, miR-122-5p and PKM2 in both HGC-27 and AGS cells. Data are presented as mean ± SD; ^***^P < 0.001 vs. NC; ^###^*P* < 0.001, ^#^*P* < 0.05 vs. si-circPHGDH. (**G** and **H**) CCK8 detection show the proliferation ability of SW579 and TPC-1 cells. The data are expressed as the mean ± SD. ^***^p < 0.001 vs. NC. (**I**-**K**) EdU detection show the proliferation ability of SW579 and TPC-1 cells. The data are expressed as the mean ± SD. ^***^p < 0.001 vs. NC. ^###^*P* < 0.001 vs. si-circPHGDH. Scale bar, 50 μm. (**L**-**N**) Transwell detection show the invasion and migration of SW579 and TPC-1 cells. The data are expressed as the mean ± SD. ^***^p < 0.001 vs. NC. ^###^*P* < 0.001 vs. si-circPHGDH. Scale bar, 50 μm

qRT-PCR data showcased that circPHGDH expression decreased post transfection with circPHGDH silencing vector. And miR-122-5p inhibitor treatment or PKM2 overexpression did not affect circPHGDH expression in SW579 and TPC-1 cells (Fig. 5A-B), saying that miR-122-5p and PKM2 were circPHGDH downstream targets. qRT-PCR data indicated that circPHGDH silencing increased miR-122-5p expression; however, PKM2 overexpression had no effect on si-circPHGDH-silenced miR-122-5p expression (Fig. 5C and D), indicating that the miR-122-5p effect was downstream from circPHGDH. Data also showcased that circPHGDH silencing decreased PKM2 expression, while miR-122-5p downregulation reversed si-circPHGDH inhibitory effect upon PKM2 expression. Post transfection with PKM2 overexpression vector, PKM2 expression incremented significantly, (Fig. 5E-F), advising that circPHGDH enhanced PKM2 expression via sponging miR-122-5p.

CCK8 (Fig. 5G and H) and EdU (Fig. 5I-K) assays showed that PKM2 overexpression or miR-122-5p suppression reversed cell proliferation of both SW579 and TPC-1 cells after silencing circPHGDH. Transwell assays to detect migration illustrated that PKM2 overexpression or miR-122-5p inhibition reversed GC cell migration regarding TPC-1 and SW579 cells post silencing circPHGDH (Fig. 5L and N).

### PKM2 overexpression or mir-122-5p suppression reversed glycolysis of PTC cells after silencing circPHGDH

**Fig. 6:**
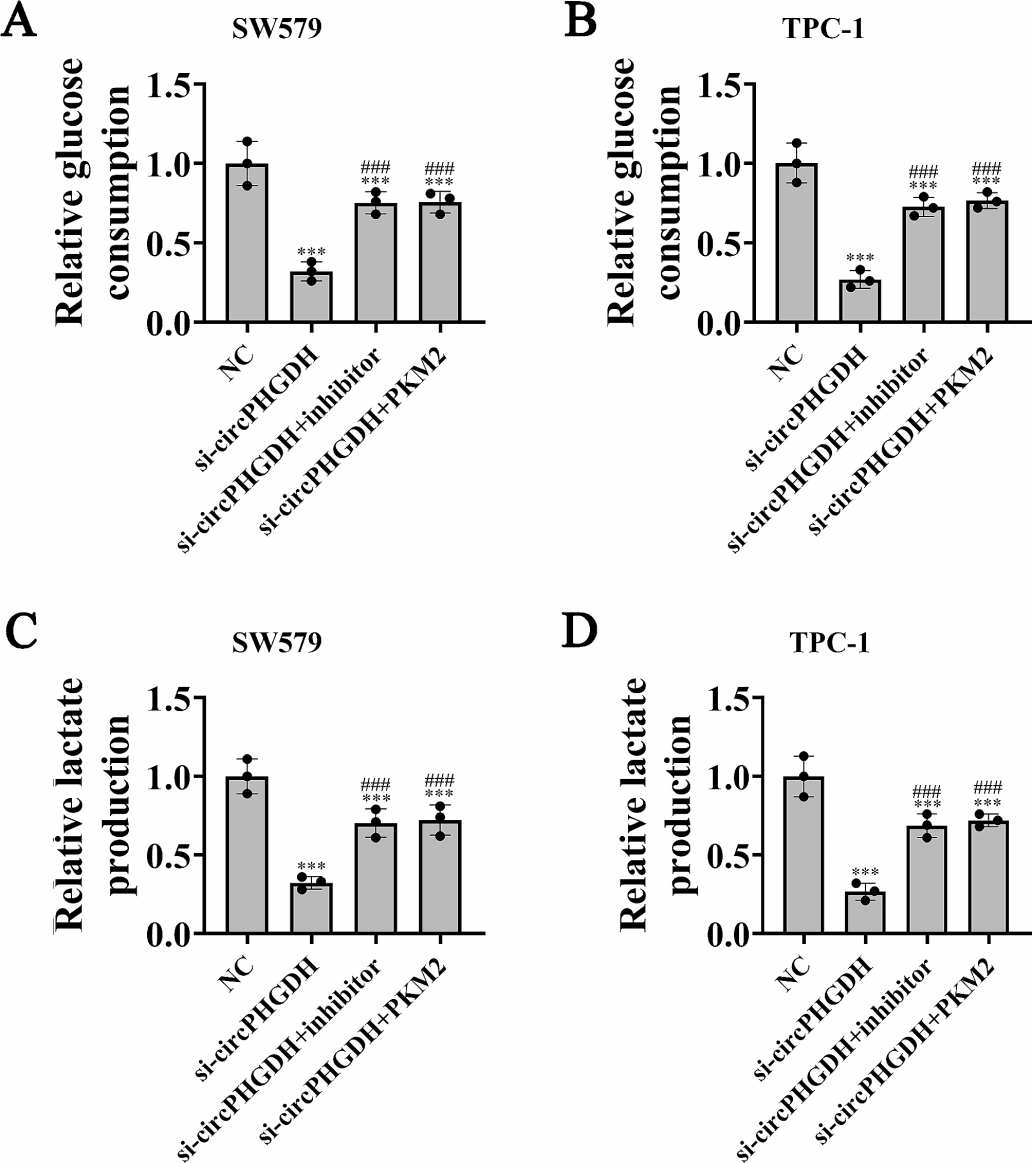
Overexpression of PKM2 or inhibit miR-122-5p reversed PTC cells glycolysis after silence circPHGDH. (**A** and **B**) Glucose consumption detection. The data are expressed as the mean ± SD. ^***^p < 0.001 vs. NC. ^###^*P* < 0.001 vs. si-circPHGDH. (**C** and **D**) Lactate production were measure in both SW579 and TPC-1 cells. The data are expressed as the mean ± SD. ^***^p < 0.001 vs. NC. ^###^*P* < 0.001 vs. si-circPHGDH.

Accumulation studies confirmed that glycolysis play an important role in regulation the malignant progression of cancer [18]. To define the effects of circPHGDH on glucose consumption as well as lactate production in PTC cells, we analyzed both SW579 and TPC-1 cells and discovered that both lactate production and glucose consumption were significantly decreased after silencing circPHGDH compared with control cells. However, PKM2 overexpression or inhibiting miR-122-5p reversed the decrease in PTC cell glycolysis by partly restoring glucose consumption and lactate production after silencing circPHGDH in TPC-1 and SW579 cells (Fig. 6A-D).

## Discussion

PTC incidence incremented steadily in last several decades. Although PTC patients generally have a favorable prognosis, tumor metastasis and invasion are main risk factors for poor prognosis [2, 19]. The Warburg effect that defined as increase in the rate of glucose uptake and preferential production of lactate, is a hallmark of cancer and provides a promising target for therapy and diagnosis. A good amount of investigations have suggested that circRNAs function importantly to regulate aerobic glycolysis in tumor progression [16, 20], but the regulatory mechanism underlying circRNAs and glycolysis in PTC are still poorly understood.

The current investigation showed that circPHGDH expression was increased in PTC tissues and cell lines. Furthermore, circPHGDH downregulation suppressed PTC proliferation, tumor growth, migration along with pulmonary metastasis in in vivo and in vitro experiments saying that circPHGDH functions in PTC progress.

Bioinformatics analysis found that miR-122-5p and PKM2 were circPHGDH downstream targets, which we confirmed through luciferase reporter assays. circPHGDH downregulation promotes miR-122-5p expression. Altered miR-122-5p expression is involved in tumorigenesis, which has been reported to function as antioncogene in gastric cancer, prostate cancer, breast cancer, NSCLC, pancreatic cancer, cervical cancer and colorectal cancer [21–25]. Previous investigations confirmed that miR-122-5p expressed at low levels in PTC, and low miR-122-5p expression promotes the PTC cell growth [26]. In the present study, our results showcased that miR-122-5p downregulation reversed inhibitory effects regarding si-circPHGDH upon PTC migration and proliferation, indicating that circPHGDH silencing inhibits PTC progression by promoting miR-122-5p expression.

Further study showed that PKM2 was an miR-122-5p downstream target, which we validated through luciferase reporter assays. circPHGDH downregulation inhibited PKM2 expression, but miR-122-5p suppression reversed inhibitory effects regarding si-circPHGDH upon PKM2 expression. Pyruvate kinase catalyzes last reactions in glycolysis via transferring high-energy phosphate from phosphoenolpyruvate to ADP, which produces pyruvate and ATP. PKM2 is dominant kinase type in proliferating and cancer cells [27]. Heterogeneity and microenvironmental influence upon cancer cells lead to different cell populations even within one simple tumor [28]. Glycolytic phenotypes do not homogeneously develop in tumors due to various PKM2 expression, which increases the glycolytic rate. Most glucose is converted to lactate with rapid ATP production [29, 30]. In present study, our lab discovered that PKM2 overexpression restored the si-circPHGDH inhibitory effects upon PTC migration and proliferation. Meanwhile, our research also found that PKM2 overexpression or inhibiting miR-122-5p reversed PTC cell glycolysis after silencing circPHGDH, partly by restoring glucose consumption and lactate production. Thus, circPHGDH silencing may inhibit PTC progression by promoting miR-122-5p and inhibiting PKM2-mediated glycolysis.

In summary, our study supports the hypothesis that downregulation of circPHGDH levels decreases PTC proliferation and invasion by target miR-122-5p/PKM2 signaling pathway regulations. These data suggest that circPHGDH is a candidate marker for PTC diagnostics and may have a promising role in PTC treatment.

## Data Availability

The data is available from the corresponding author on reasonable request.

## References

[1] Lim H, Devesa SS, Sosa JA, Check D, Kitahara CM (2017). Trends in thyroid cancer incidence and mortality in the United States, 1974–2013. JAMA.

[2] Jiang C, Cheng T, Zheng X, Hong S, Liu S, Liu J, Wang J, Wang S (2018). Clinical behaviors of rare variants of papillary thyroid carcinoma are associated with survival: a population-level analysis. Cancer Manag Res.

[3] Ferlay J, Shin HR, Bray F, Forman D, Mathers C, Parkin DM (2010). Estimates of worldwide burden of cancer in 2008: Globocan 2008. Int J Cancer.

[4] Haugen BR, Alexander EK, Bible KC, Doherty GM, Mandel SJ, Nikiforov YE, Pacini F, Randolph GW, Sawka AM, Schlumberger M, Schuff KG, Sherman SI, Sosa JA, Steward DL, Tuttle RM, Wartofsky L (2016). 2015 American thyroid association management guidelines for adult patients with thyroid nodules and differentiated thyroid cancer: the American thyroid association guidelines task force on thyroid nodules and differentiated thyroid cancer. Thyroid.

[5] Du G, Ma R, Li H, He J, Feng K, Niu D, Yin D (2021). Increased expression of hsa_circ_0002111 and its clinical significance in papillary thyroid cancer. Front Oncol.

[6] Zhu J, Wang Y, Yang C, Feng Z, Huang Y, Liu P, Chen F, Deng Z. Circ-psd3 promoted proliferation and invasion of papillary thyroid cancer cells via regulating the mir-7-5p/mettl7b axis. J Recept Signal Transduct Res 2021:1–10. 10.1080/10799893.2021.1910706 33858297

[7] Yang Y, Ding L, Li Y, Xuan C (2020). Hsa_circ_0039411 promotes tumorigenesis and progression of papillary thyroid cancer by mir-1179/abca9 and mir-1205/mta1 signaling pathways. J Cell Physiol.

[8] Qi Y, He J, Zhang Y, Wang L, Yu Y, Yao B, Tian Z. Circular rna hsa_circ_0001666 sponges mir3305p, mir193a5p and mir326, and promotes papillary thyroid carcinoma progression via upregulation of etv4. Oncol Rep 2021;45. 10.3892/or.2021.8001 PMC7934216 33760216

[9] Liu L, Yan C, Tao S, Wang H (2020). Circ_0058124 aggravates the progression of papillary thyroid carcinoma by activating lmo4 expression via targeting mir-370-3p. Cancer Manag Res.

[10] Wang M, Chen B, Ru Z, Cong L (2018). Circrna circ-itch suppresses papillary thyroid cancer progression through mir-22-3p/cbl/beta-catenin pathway. Biochem Biophys Res Commun.

[11] Granados-Riveron JT, Aquino-Jarquin G (2016). The complexity of the translation ability of circrnas. Biochim Biophys Acta.

[12] Nana-Sinkam SP, Croce CM (2014). Microrna regulation of tumorigenesis, cancer progression and interpatient heterogeneity: towards clinical use. Genome Biol.

[13] Li Z, Huang C, Bao C, Chen L, Lin M, Wang X, Zhong G, Yu B, Hu W, Dai L, Zhu P, Chang Z, Wu Q, Zhao Y, Jia Y, Xu P, Liu H, Shan G (2015). Exon-intron circular rnas regulate transcription in the nucleus. Nat Struct Mol Biol.

[14] Jiang B, Chen Y, Xia F, Li X (2021). Ptcsc3-mediated glycolysis suppresses thyroid cancer progression via interfering with pgk1 degradation. J Cell Mol Med.

[15] Wang JM, Jiang JY, Zhang DL, Du X, Wu T, Du ZX (2021). Hyou1 facilitates proliferation, invasion and glycolysis of papillary thyroid cancer via stabilizing ldhb mrna. J Cell Mol Med.

[16] Zuo XM, Sun HW, Fang H, Wu Y, Shi Q, Yu YF (2021). Mir-4443 targets trim14 to suppress metastasis and energy metabolism of papillary thyroid carcinoma (ptc) in vitro. Cell Biol Int.

[17] Zhao L, Wei J, Wang S, Lang T, Shi X, Shan Z, Teng W (2020). Beta-elemene inhibits differentiated thyroid carcinoma metastasis by reducing cellular proliferation, metabolism and invasion ability. Ann Transl Med.

[18] Shi T, Ma Y, Cao L, Zhan S, Xu Y, Fu F, Liu C, Zhang G, Wang Z, Wang R, Lu H, Lu B, Chen W, Zhang X (2019). B7-h3 promotes aerobic glycolysis and chemoresistance in colorectal cancer cells by regulating hk2. Cell Death Dis.

[19] Song E, Jeon MJ, Oh HS, Han M, Lee YM, Kim TY, Chung KW, Kim WB, Shong YK, Song DE, Kim WG (2018). Do aggressive variants of papillary thyroid carcinoma have worse clinical outcome than classic papillary thyroid carcinoma?. Eur J Endocrinol.

[20] Ma J, Kan Z (2021). Circ_0137287 suppresses cell tumroigenesis and aerobic glycolysis in papillary thyroid carcinoma through mir-183-5p/ppp2r2a axis. Cytotechnology.

[21] Jiao Y, Zhang L, Li J, He Y, Zhang X (2021). Exosomal mir-122-5p inhibits tumorigenicity of gastric cancer by downregulating git1. Int J Biol Markers.

[22] Li C, Qin F, Wang W, Ni Y, Gao M, Guo M, Sun G. Hnrnpa2b1-mediated extracellular vesicles sorting of mir-122-5p potentially promotes lung cancer progression. Int J Mol Sci 2021;22. 10.3390/ijms222312866 PMC8658035 34884671

[23] Mazza T, Gioffreda D, Fontana A, Biagini T, Carella M, Palumbo O, Maiello E, Bazzocchi F, Andriulli A, Tavano F (2020). Clinical significance of circulating mir-1273 g-3p and mir-122-5p in pancreatic cancer. Front Oncol.

[24] Ding FN, Gao BH, Wu X, Gong CW, Wang WQ, Zhang SM (2019). Mir-122-5p modulates the radiosensitivity of cervical cancer cells by regulating cell division cycle 25a (cdc25a). FEBS Open Bio.

[25] Chen J, Wu W, He X, Jia L, Yang J, Si X, Yu K, Li S, Qiu Y, Xu K, Yin P, Cao Y, Li Q, Li W (2021). Exosomal mir-122-5p is related to the degree of myelosuppression caused by chemotherapy in patients with colorectal cancer. Cancer Manag Res.

[26] Hu N, Tian Y, Song Y, Zang L. Mir1225p suppresses the oncogenesis of ptc by inhibiting dusp4 expression. Mol Med Rep 2021;23. 10.3892/mmr.2021.12007 PMC7986011 33760201

[27] Zahra K, Dey T, Ashish, Mishra SP, Pandey U (2020). Pyruvate kinase m2 and cancer: the role of pkm2 in promoting tumorigenesis. Front Oncol.

[28] Wang X, Zhang H, Yang H, Bai M, Ning T, Deng T, Liu R, Fan Q, Zhu K, Li J, Zhan Y, Ying G, Ba Y (2020). Exosome-delivered circrna promotes glycolysis to induce chemoresistance through the mir-122-pkm2 axis in colorectal cancer. Mol Oncol.

[29] Wang JZ, Zhu W, Han J, Yang X, Zhou R, Lu HC, Yu H, Yuan WB, Li PC, Tao J, Lu Q, Wei JF, Yang H (2021). The role of the hif-1alpha/alyref/pkm2 axis in glycolysis and tumorigenesis of bladder cancer. Cancer Commun (Lond).

[30] Yu W, Yang Z, Huang R, Min Z, Ye M (2019). Sirt6 promotes the warburg effect of papillary thyroid cancer cell bcpap through reactive oxygen species. Onco Targets Ther.

